# COVID-19 and shrinking civic spaces: patterns and consequences

**DOI:** 10.1007/s42597-020-00038-w

**Published:** 2020-10-02

**Authors:** Felix S. Bethke, Jonas Wolff

**Affiliations:** Peace Research Institute Frankfurt (PRIF), Baseler Straße 27–31, 60329 Frankfurt am Main, Germany

**Keywords:** Civil society, Human rights, Freedom of assembly, Repression, Autocratization, Zivilgesellschaft, Menschenrechte, Versammlungsfreiheit, Repression, Autokratisierung

## Abstract

In response to the COVID-19 pandemic, a majority of countries worldwide have introduced severe limitations on the freedom of assembly, if not an outright lockdown, in many cases complemented by restrictions on further civil and political rights. Although restrictions were generally considered necessary to save lives and protect health care systems from overburdening, they also pose the risk of government overreach, that is, governments may use the pandemic as a convenient opportunity and justification to impose restrictions for political purposes. In this sense, COVID-19 may give yet another substantial boost to a global trend that has been unfolding since the early 2000s: the shrinking of civic spaces, which is characterized by an increase in government restrictions that target civil society actors and limit their freedoms of assembly, association, and expression. The aim of the paper is to assess civic space restrictions that have been imposed in response to the COVID-19 pandemic with a view to exploring their immediate consequences as well as their potential mid-term implications for civil society organizations in general and contentious civic activism in particular. We do so by, first, providing evidence from multiple data sources about the global spread of COVID-19-related restrictions over time and across countries. Second, we identify key dynamics at work in order to assess the immediate consequences and the potential mid-term implications of these restrictions. These dynamics are illustrated by looking at experiences from individual countries (including Cambodia, Germany, Hungary, and Lebanon).

**Electronic supplementary material:**

The online version of this article (10.1007/s42597-020-00038-w) contains supplementary material, which is available to authorized users.

## Abstract (de)

Als Reaktion auf die COVID-19-Pandemie haben Staaten weltweit die Versammlungsfreiheit stark eingeschränkt bis hin zu kompletten Lockdown-Maßnahmen. In vielen Fällen erfolgten dabei weitere Einschränkungen bürgerlicher und politischer Rechte. Obwohl diese Einschränkungen generell als notwendig erachtet wurden, um Leben zu retten und die Gesundheitssysteme vor Überlastung zu schützen, bergen sie auch das Risiko von government overreach, d.h. Regierungen können die Pandemie als willkommene Gelegenheit und Rechtfertigung nutzen, um zivile und politische Menschenrechte aus politischem Kalkül einzuschränken. Somit könnte COVID-19 einem globalen Trend verstärken, der sich seit Anfang der 2000er Jahre beobachten lässt: das Schrumpfen zivilgesellschaftlicher Handlungsspielräume bzw. die weltweite Zunahme staatlicher Restriktionen, welche zivilgesellschaftliche Akteure und ihre Versammlungs-, Vereinigungs-, und Meinungsfreiheit einschränken. Dieser Artikel analysiert die als Reaktion auf die COVID-19-Pandemie eingeführten Restriktionen zivilgesellschaftlicher Handlungsspielräume hinsichtlich ihrer unmittelbaren Folgen sowie ihrer möglichen mittelfristigen Auswirkungen auf zivilgesellschaftliche Organisationen im Allgemeinen und zivilgesellschaftlichen Aktivismus im Besonderen. Erstens rekonstriueren wir mittels verschiedener Daten die globale Verbreitung zivilgesellschaftlicher Restriktionen im Kontext der Pandemie im Zeitverlauf. Zweitens identifizieren wir zentrale Dynamiken und Mechanismen, um die unmittelbaren Folgen und die möglichen mittelfristigen Auswirkungen dieser Einschränkungen abzuschätzen. Die identifizierten Dynamiken werden anhand von Erfahrungen aus einzelnen Ländern (u.a. Kambodscha, Deutschland, Ungarn und Libanon) veranschaulicht.

## Introduction

Starting in early February 2020, a rapidly increasing number of governments began to impose severe restrictions on core civic freedoms as a means to contain the COVID-19 pandemic. Until early April 2020, a majority of countries worldwide had introduced severe limitations on the freedom of assembly, if not an outright lockdown, in many cases complemented by restrictions on further civil and political rights. Generally speaking, international human rights organizations and experts recognized that even such drastic restrictions on human rights can be legitimate responses to the spread of the coronavirus (Amnesty International 2020; HRW 2020a; OHCHR 2020).^1^ Yet, they simultaneously warned of the serious risk that governments might make use of the very emergency situation for political purposes. In fact, as Doug Rutzen and Nikhil Dutta (2020) warned early on: “Pandemics are fertile breeding grounds for governmental overreach”.

This risk is particularly high because the COVID-19 pandemic has hit the world at a time when a significant number of countries were already moving in the direction of what has been dubbed “closing” or “shrinking civic spaces”. Since the early 2000s, politicians, civil society activists and scholars have observed a worldwide increase in government restrictions targeting civil society activists and organizations (CSOs) and limiting their space, autonomy and/or capacity (see Carothers and Brechenmacher 2014; Poppe and Wolff 2017).^2^ While this phenomenon is complex and has many sources, another exceptional event with global repercussions played an important role in the current global diffusion of civic space restrictions: The terrorist attacks of 11 September 2001 and the subsequent “global war on terror” provided governments around the world “with a convenient discourse and justification for tightening their hold over NGOs and their opponents” (Howell et al. 2008, p. 87). In addition, international counterterrorism measures—and namely the attempts by the Financial Action Task Force (FATF) to limit terrorist financing by strengthening the oversight of non-profit organizations—directly pushed governments towards adopting more restrictive NGO (funding) rules in many places.^3^

Against this background, we assess civic space restrictions that have been imposed in response to the COVID-19 pandemic in two ways. First, we provide evidence from multiple data sources about the global spread of COVID-19-related restrictions over time and across countries. Second, we discuss the immediate consequences and mid-term implications of these restrictions by identifying key dynamics at work in individual countries (including Cambodia, Germany, Hungary, and Lebanon).

Our findings show that COVID-19 has provoked an unprecedented, global wave of civic space restrictions. Across the board, these restrictive measures have limited the freedom of assembly but, in several countries, governments have also constrained the freedom of expression. With a view to the potential mid-term consequences, our analysis suggests ambivalent developments. On the one hand, in countries that had already been characterized by processes of de-democratization or autocratization COVID-19 seems to reinforce these dynamics. On the other hand, however, we argue that the predominant forms of restrictions adopted in response to the pandemic do not lend themselves to policies that deliberately target specific groups (e.g. CSOs), as in the case of in counter-terrorism policies. Instead, COVID-19-related restrictions affect the general population and are, therefore, more likely to cause broad-based resistance. This makes them more difficult to sustain over time, but can also provoke escalating conflict.

With a view to the overall research on shrinking civic spaces, our contribution highlights the relevance of justifications put forward by governments when imposing civic space restrictions (see also Poppe and Wolff 2017). More specifically, this article adds to the role of idiosyncratic events, external shocks and emergency situations that shape the spread of, and the controversies over, civic space restrictions. As opportunities, they enable governments to justify extraordinary measures that would otherwise provoke (much more) resistance. At the same time, as these restrictions are justified as response to a particular threat, the very type of emergency also constrains the plausible range and type of measures. While in the overall debate 9/11 and its implications for the spread of shrinking civic spaces have received a lot of attention (see Carothers and Brechenmacher 2014; Howell et al. 2008; Poppe and Wolff 2017), existing comparative studies rather focus on general factors (e.g. levels of foreign aid or commitment to human rights treaties) and recurring events (such as elections) to explain the phenomenon (see, for instance, Bakke et al. 2020; Dupuy et al. 2016) and have yet to systematically include individual, but salient events and crises as potential explanation.

## Civic space restrictions amid the COVID-19 pandemic: an overview

To explore the scope of civic space restrictions and their implementation over time, we focus on policies that imply constraints on three key civil rights: the freedoms of assembly, association, and expression. Based on data provided by the COVID-19 Civic Freedom Tracker (ICNL and ECNL 2020), Fig. 1 shows the timing and frequency of these different types of restrictions that countries imposed between February and July 2020.^4^

**Fig. 1:**
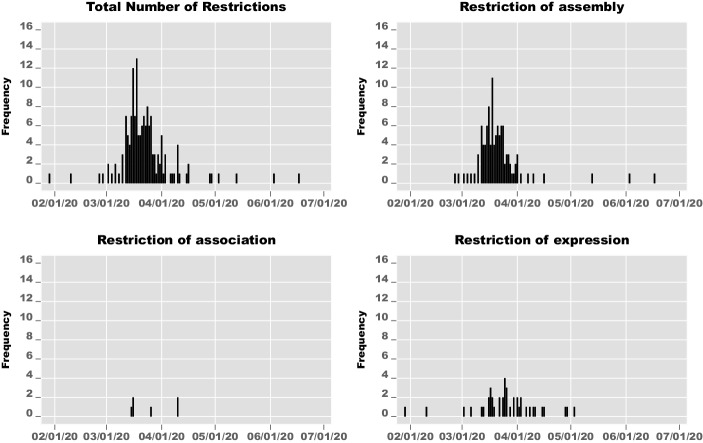
Introduction of civic space restrictions by countries over time (February–July 2020)

Fig. 1 illustrates the rapid pace with which governments responded to the pandemic with restrictive policies. Most restrictions were implemented during the last two weeks of March 2020. Moreover, it becomes clear that the global scope of civic space restrictions has reached unprecedented levels. Based again on the COVID-19 Civic Freedom Tracker, Table 1 breaks down the frequencies of different types of restrictions across world regions.

**Table 1:** Types of restrictions across world regions (February–July 2020)

Region	Assembly	Association	Expression
Africa	34	0	11
Americas	18	2	4
Asia	24	3	20
Europe	26	0	6
Oceania	6	1	1
Worldwide	108	6	42

As can be seen, restrictions occurred across all world regions and focus mostly on assembly rights (e.g. by banning mass gatherings of citizens). For the time period of February to July 2020 the COVID-19 Civic Freedom Tracker records 108 countries restrictions of the freedom of assembly. In contrast, the data show that only few countries implemented restrictions targeting the freedom of association. These six cases mostly refer to governments that enacted limitations for CSOs as part of emergency laws to respond to the pandemic. Restrictions targeting freedom of expressions occurred more frequently, i.e. in 42 countries. Most of these restrictions either limited press freedom or enacted laws against the spread of COVID-19-related rumors. Lastly, it should be noted that restrictions often come as a package, i.e. the government issues a bill for emergency restrictions to extend its powers, which also restrict civic freedoms at the same time.

These numbers confirm that civic space restrictions in response to COVID-19 have been both widespread and far-reaching. Yet, they do not show the extent of governmental overreach. In general, the most common policy (restrictions on freedom of assembly) is closely related to pandemic control, as it directly enables the government to slow down the spread of the virus by enforcing “physical distancing”. The contribution to pandemic control of restrictions of association and expression is less direct, but even here cannot be ruled out categorically (e.g., when the spread of conspiracy theories undermines efforts to ensure “physical distancing” or the wearing of face masks). In order to systematically assess the appropriateness of COVID-19-related measures, human rights organizations and experts have emphasized that government restrictions should be in line with internationally recognized human rights standards, i.e. they should be precise, transparent, proportionate, of limited duration, and subject to legislative and judicial oversight (see Amnesty International 2020; HRW 2020a; OHCHR 2020; Rutzen and Dutta 2020). We analyze this aspect of COVID-19-induced civic space restrictions with data from the V‑Dem Institute’s Pandemic Backsliding Project (PanDem) dataset, which uses expert coding to identify the extent to which “government responses to the Covid-19 pandemic violated democratic standards for emergency measures” between March and June 2020 (Edgell et al. 2020). According to this data, more than half of the 146 countries assessed have seen some (66) or even major (16) violations of such standards. Only 34 governments managed to respond to the pandemic without violations.^5^ Major violations have been recorded in otherwise most different countries. In addition to five African and six Asian countries, two are European (Hungary, Serbia) and two from Latin America and the Caribbean (El Salvador, Haiti). In terms of regime type, according to V‑Dem, countries range from electoral democracies (e.g., El Salvador and India) all the way to closed autocracies (e.g., China or Saudi Arabia). Notably, major violations occurred mainly in countries that had been characterized by shrinking civic spaces already before the pandemic hit (such as China, Egypt, Hungary or India).^6^

Finally, we explore whether governments have started to withdraw restrictions due to decreasing pandemic hazard in some world regions. For the time period January until the end of July 2020, Fig. 2 describes the prevalence and severity of restrictions of public gatherings, drawing on data provided by the Oxford Covid-19 Government Response Tracker (Hale et al. 2020).^7^

**Fig. 2:**
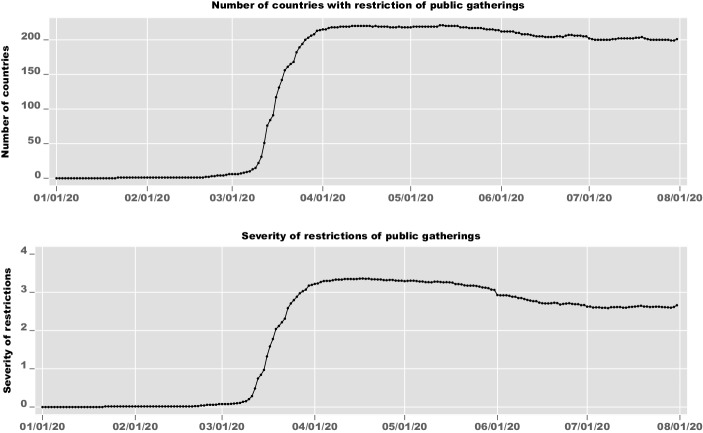
Endurance, withdrawal, and severity of restrictions over time

The upper panel describes the number of countries where at least some restrictions of public gatherings are active. The lower panel describes the average severity of restrictions. As shown in Fig. 2, the number of countries that have restrictions of public gatherings in place declines only marginally until the end of the time series. However, the severity of active restrictions starts to decline substantially in May 2020. Thus, for the time being, governments increasingly relax restrictions, but are not willing to abandon them amid the pandemic hazard.

## Consequences for civil society organizations and protest movements

We identify three key dynamics through which responses to the COVID-19 pandemic affect civic space. These dynamics will be illustrated by briefly discussing experiences from four cases, which represent the range of violations of democratic standards as measured by the above-mentioned PanDem dataset. First, we discuss how COVID-19-related restrictions shape civic protest and, in particular, discuss the case of Lebanon (minor violations). Second, we turn to consequences for the freedom of expression, using the case of Cambodia (some violations) as an example. Third, we assess the potential mid-term impact on civic space and, here, contrast the experiences of Hungary (major violations) and Germany (no violations).

The most visible immediate consequence of the emergency measures has been the far-reaching closure of the physical civic space to assemble. In several countries, this closure was enforced by the military and/or through the use of violent repression. According to PanDem data, 57 countries across all world regions have seen the military being involved in enforcing the restrictions on the freedoms of movement and assembly. Cases of violent repression have been reported, for instance, from Kenya, Nigeria and Uganda (Kagumire 2020). The immediate result has been “an unprecedented fall in protest activity around the world” (Metternich 2020, p. 1), abruptly interrupting the global wave of protests that had characterized recent years. In fact, for governments in countries such as Chile and Colombia, Algeria and Lebanon, China/Hong Kong and India, the pandemic arguably provided a welcome opportunity to stop the mass protests that had challenged them in the months before. Yet, as data from the Carnegie Global Protest Tracker suggests, this reduction in protest activities will likely remain a “temporary lull” only: Even as difficult conditions for, if not an outright prohibition of demonstrations persisted, mass protests quickly “have begun to return at a notable rate and scale” in many places including in Brazil, Ecuador, Hong Kong, Iraq, Lebanon, Mali, the US and a number of European countries (Carothers and Wong 2020).

Lebanon is a good case to illustrate this dynamic. On 16 March 2020, Lebanon declared a state of emergency, which included a general prohibition of private and public gatherings. While Lebanon’s emergency measures, according to PanDem, mostly respected democratic standards, the government did deploy the military to enforce the lockdown (ICNL 2020a, p. 3). In the preceding months, a powerful protest movement had put continuous pressure on the political regime, forcing the resignation of Prime Minister Saad Hariri in October 2019, but with the lockdown in mid-March the protests “petered out” (Hubbard and Saad 2020). In fact, the authorities used the opportunity of the lockdown, as security forces reportedly removed protesters’ tents, “arresting the remaining few protestors and opening the streets which had been blocked for months” (Safwan 2020). Yet, renewed anti-government protests quickly emerged, driven by the aggravating economic crisis (see Hubbard and Saad 2020; Safwan 2020). The situation escalated further after the large-scale explosions that occurred at the port of the city of Beirut on 4 August 2020. Afterwards, protests gained new momentum and led to the occupation of ministry buildings by citizens and violent clashes between demonstrators and security forces.

In addition to the broad limitations on the freedoms of movement and assembly, more targeted restrictions on the freedom of expression in a series of countries have negatively affected the public sphere as provided by traditional and online (including “social”) media. According to the COVID-19 Civic Freedom Tracker, governments in 28 countries^8^ introduced legal measures that prohibit the dissemination of supposedly false or misleading information, and in several countries—e.g. in Cambodia, Iran, Malaysia, Sri Lanka, Tunisia and Turkey—people have been arrested on such grounds (ICNL and ECNL 2020).

In the case of Cambodia, according to the International Federation of Journalists (IFJ 2020), emergency legislation approved on 10 April 2020 granted “the government powers to monitor communications, control media and prohibit or limit the distribution of information deemed to trigger public fear and damage national security”. Already before this legislation came into effect, Human Rights Watch (HRW 2020b) had documented several cases in which people, including journalists and members of Cambodia’s dissolved opposition party, had been arrested on charges of spreading false information.

In order to explore the potential mid-term impact of COVID-19-related restrictions on the further evolution of civic spaces around the world, three questions are of particular relevance. The first question is whether restrictive measures adopted in response to the pandemic remain in place beyond the emergency situation. Second, even if restrictions expire or are repealed, do persisting changes to the legal order and/or the public discourse facilitate the renewed imposition and/or justification of restrictive measures in the future? Third, does the overall response to COVID-19 contribute to a broader process of autocratization of the political regime, which involves—among other things—a (further) shrinking of the civic space?

Hungary is a case which combines negative developments in all three regards. On 11 March 2020, the government of Prime Minister Viktor Orbán declared a national “state of danger” and, on 30 March, parliament adopted the Coronavirus Protection Act, which indefinitely extended the emergency state, granting the government virtually unlimited powers to rule by decree. In addition, the law defines the spread of false or distorted information that might obstruct the emergency measures of the government as crime punishable with up to five years of prison (Walker 2020). In the course of June 2020, the “state of danger” was actually lifted. Yet, on the one hand, some of the restrictive measures have actually remained in place—such as the penal prohibition of “scaremongering” through misinformation in situations of public emergency, which was included in the country’s Criminal Code. On the other, legal changes enable the government to adopt, in response to the current or any future health crisis, all measures deemed necessary without parliamentary approval, including restrictions on the freedom of movement or the freedom of assembly (see ECPMF 2020; Hungarian Helsinki Committee et al. 2020). Overall, these persisting changes to the legal order further contribute to the gradual process of autocratization that Hungary has been experiencing since 2010 (Maerz et al. 2020, p. 7–8).

Existing comparative assessments of emergency measures adopted in response to the COVID-19 pandemic also show that “many governments have demonstrated that it is possible to safeguard rights while effectively countering the virus” (ICNL 2020b, p. 1). As previously mentioned, the PanDem data indicates that 64 countries (44%) registered no or only minor violations of democratic standards. In fact, as the case of Germany suggests, the legacy of the (first wave of) anti-COVID-19 measures might even be positive for civic space under specific circumstances. Initially, in line with the nation-wide “contact ban” which proscribed public gatherings of more than two people, most German states (*Länder*) generally suspended the freedom of assembly. While many local authorities implemented this norm by not permitting any demonstrations (and, in some occasions, even by forcefully dissolving unauthorized protests), the Constitutional Court ruled on 16 April that “health concerns linked to the coronavirus pandemic are no grounds for a general ban on demonstrations”, which would violate “the constitutional right to assembly” (Nasr 2020). This sets an important legal precedence. As a consequence, it is highly unlikely that, in case of a renewed tightening of the anti-COVID-19 measures in Germany, authorities would again issue a blanket ban on protest.

## Conclusions

The recent global developments analyzed in this contribution have not only brought about an unprecedented wave of civic space restrictions all around the world. They also entail the risk that governmental responses to the COVID-19 pandemic may give another lasting boost to the spread of shrinking civic spaces, a phenomenon that has been observed since the early 2000s. As we noted in the introduction, the experience with 9/11, the subsequent “global war on terror” and the role that counterterrorism measures and the overarching counterterrorism discourse played in the introduction and justification of civic space restrictions represents a serious warning in this regard. Our analysis, on the one hand, confirms that the risk of government overreach is real and that COVID-19 already seems to be contributing to a further de-democratization or autocratization of countries that have already been on this trajectory before the pandemic hit (see also Edgell et al. 2020). On the other hand, the more precise assessment of the specific forms of civic space restrictions introduced in response to COVID-19 also reveals grounds for cautious optimism. In the aftermath of 9/11, counterterrorism measures and discourses, in particular, gave rise to *targeted* restrictions of the freedom of *association* of certain CSOs that were regarded, or presented, as particularly vulnerable to being used for supporting terrorist endeavors (e.g., foreign funded NGOs or Muslim charities). By contrast, restrictions in the context of the COVID-19 pandemic not only but primarily focus on *overall* restrictions of the freedom of *assembly*. In fact, when compared to counterterrorism, anti-pandemic policies are much less well suited to justifying targeted, politically motivated restrictions. As the current resurgence of mass protests in many countries suggests, indiscriminate COVID-19-related restrictions are likely to provoke broad-based resistance and are, therefore, much more difficult to sustain over time.

In sum, our analysis suggests that the overall result of the political response to the COVID-19 pandemic will not so much be a direct and lasting (further) closure of the civic space. Rather, the pandemic, the political response to COVID-19 and the overall socioeconomic consequences tend to aggravate existing conflicts and controversies—with indeterminate outcomes for the evolution of both civic spaces and the overall shape of political regimes. Yet, these results are certainly preliminary only and more systematic comparative research is needed. In particular, we see three main areas for further research. First, additional and continuous research should evaluate and monitor the lasting consequences of COVID-19-related restrictions for civic freedoms, especially whether and why restrictions endure even after the immediate emergency situation. Second, important questions concern the responses to COVID-19-related restrictions by civil society actors, e.g., how CSOs change and adapt to the new circumstances and how organizations and movements mobilize against government overreach. Third, with a view to the broader research agenda on shrinking civic space, it would be promising to systematically compare COVID-19-related developments with civic space restrictions in the wake of 9/11, in order to identify the potentially specific logics of emergency-induced episodes.

## Caption Electronic Supplementary Material

The Electronic Supplementary Material contains the raw data files used in the article (i.e. “full_data_V3” – Varieties of Democracy, PanDem dataset, “icnl4” – ICNL COVID-19 Civic Freedom Tracker data, and “OxCGRT_latest” – Oxford Covid-19 Government Response Tracker dataset) along with the replication code (“replication code.do”) to create tables and figures.

## Footnotes

1 Under international human rights law, which includes the right to health, governments are in fact obliged to do their best to prevent, treat and control “epidemic, endemic, occupational and other diseases” (UN 1966).

2 These restrictions range from administrative barriers that hamper the registration of organizations, to “NGO laws” that delegitimize or limit foreign funding, measures of censorship, surveillance or intimation to the judicial prosecution or physical threatening of CSOs and activists.

3 According to Carothers and Brechenmacher (2014, p. 29), in the wake of 9/11, more than 140 governments “passed new counterterrorism legislation […], often in response to U.S. pressure, UN Security Council resolutions, and the counterterrorism guidelines developed by the Financial Action Task Force (FATF)”.

4 The COVID-19 Civic Freedom Tracker monitors government responses to the pandemic that affect civic freedoms and human rights. Responses include laws, orders, regulations, policies, and practices implemented by governments (ICNL and ECNL 2020). We scraped the data from the ICNL website as of September 1st 2020. For the analysis, we considered the first introduction of each type of restriction for each country. Thus, subsequent adjustments which relaxed, extended or exacerbated restrictions were not counted. For the total number of restrictions reported in the upper-left panel of Fig. 1, countries were counted multiple times if they introduced different types of restrictions.

5 These numbers were calculated using the “Alternative Pandemic Democratic Violations Index” from the PanDem dataset.

6 A second group of countries in this category had been characterized by a relatively stable level of severe civic space restrictions in the context of closed authoritarian regimes (e.g. Saudia Arabia) and/or violent conflict (e.g. DR Congo). Notable exceptions include, for instance, El Salvador (which had not seen a trend of shrinking civic space before the pandemic) and Ethiopia (which had recently seen improvements that had partially reversed a previous wave of civic space restrictions).

7 The database codes restriction of public gatherings on a scale from zero to four. A score of zero indicates no restrictions. A score of one means only very large gatherings of more than 1000 people are forbidden. Policies that restrict gatherings between 101–1000 people receive a score of two. Restrictions of gatherings between 11–100 people and of 10 people or less receive a score of three and four respectively. For the upper panel, we considered the number of countries that implemented any restrictions of gatherings, i.e. a score of one or higher (numbers differ from those reported by ICNL and ECNL due to differences in coding rules). For the lower panel, we calculated the average score across countries over time.

8 Data was compiled from the ICNL website as of September 1st, 2020. We considered legal restriction of freedom of expression, which explicitly refer to the punishment of dissemination of COVID-19 related information. Countries that introduced respective laws, regulation and orders include: Algeria, Armenia, Azerbaijan, Bolivia, Bosnia and Herzegovina, Botswana, Cambodia, Chad, China, Ethiopia, Hungary, India, Jordan, Kenya, Lesotho, Moldova, Morocco, North Macedonia, Palestine, Philippines, Russia, Serbia, Solomon Islands, South Africa, Thailand, United Arab Emirates, Uzbekistan, and Zimbabwe.

## References

[CR1] Amnesty International. 2020. Responses to COVID-19 and states’ human rights obligations: preliminary observations. https://www.amnesty.org/en/documents/pol30/1967/2020/en. Accessed 6 July 2020.

[CR2] Bakke, Kristin M., J. Mitchell Neil, and Hannah M. Smidt. 2020. When states crack down on human rights defenders. *International Studies Quarterly* 64(1):85–96.

[CR3] Carothers, Thomas, and Saskia Brechenmacher. 2014. *Closing space. Democracy and human rights support under fire*. Washington, DC: Carnegie Endowment for International Peace.

[CR4] Carothers, Thomas, and David Wong. 2020. Global protests start to return. https://carnegieendowment.org/2020/06/30/global-protests-start-to-return-pub-82225. Accessed 6 July 2020.

[CR5] Dupuy, Kendra, James Ron, and Aseem Prakash. 2016. Hands off my regime! Governments’ restrictions on foreign aid to non-governmental organizations in poor and middle-income countries. *World Development* 84:299–311.

[CR7] ECPMF (European Centre for Press & Media Freedom).. 2020. Hungary’s two pandemics: COVID-19 and attacks on media freedom. https://www.ecpmf.eu/hungarys-two-pandemics-covid-19-and-attacks-on-media-freedom. Accessed 8 July 2020.

[CR6] Edgell, Amanda B., Anna Lührmann, Seraphine F. Maerz, Jean Lachapelle, Sandra Grahn, Ana Flavia Good God, Martin Lundstedt, Natalia Natsika, Palina Kolvani, and Shreeya Pillai, Abdalhadi Alijla, Tiago Fernandes, Hans Tung, Matthew Wilson and Staffan I. Lindberg. 2020. Pandemic Backsliding: Democracy During Covid-19 (PanDem), Version 3. Varieties of Democracy (V-Dem) Institute. http://www.v-dem.net/en/our-work/research-projects/pandemic-backsliding. Accessed: 3 July 2020.

[CR8] Hale, Thomas, Sam Webster, Anna Petherick, Toby Phillips, and Beatriz Kira. 2020. *Oxford COVID-19 government response tracker*. : Blavatnik School of Government, Oxford. 10.1038/s41562-021-01079-8 33686204

[CR9] Howell, Jude, Armine Ishkanian, Ebenezer Obadare, Hakan Seckinelgin, and Marlies Glasius. 2008. The Backlash Against Civil Society in the Wake of the Long War on Terror. *Development in Practice* 18(1):82–93.

[CR11] HRW. 2020b. Cambodia: Covid-19 spurs bogus news’ arrests. https://www.hrw.org/news/2020/04/29/cambodia-covid-19-spurs-bogus-fake-news-arrests. Accessed 8 July 2020.

[CR12] Hubbard, Ben, and Hwaida Saad. 2020. Lebanon’s Currency Plunges, and Protesters Surge Into Streets. *The New York Times, *11 June 2020. https://www.nytimes.com/2020/06/11/world/middleeast/lebanon-protests.html. Accessed 8 July 2020.

[CR10] Human Rights Watch. 2020a. Respect rights in COVID-19 response: recommendations for governments in addressing pandemic. https://www.hrw.org/news/2020/03/19/respect-rights-covid-19-response. Accessed 6 July 2020.

[CR13] Hungarian Helsinki Committee, Hungarian Civil Liberties Union, and Amnesty International Hungary.. 2020. Never-ending story? Rapid analysis of the bill on terminating the state of danger (T/10747) & the bill on transitional provisions related to the termination of the state of danger (T/10748). https://www.helsinki.hu/en/never-ending-story. Accessed 8 July 2020.

[CR14] ICNL (International Center for Not-For-Profit Law). 2020a. Top trends: COVID-19 and civic space. https://www.icnl.org/post/analysis/top-trends-covid-19-and-civic-space. Accessed 8 July 2020.

[CR16] ICNL and ECNL (European Center for Not-for-Profit Law).. 2020. COVID-19 civic freedom tracker. https://www.icnl.org/covid19tracker. Accessed 7 July 2020.

[CR15] ICNL. 2020b. Coronavirus & civic space: positive government practices in responding to COVID-19. https://www.icnl.org/post/analysis/positive-government-responses-to-covid-19. Accessed 8 July 2020.

[CR17] IFJ (International Federation of Journalists). 2020. Cambodia: parliament approves “state emergency” law Amid the pandemic. https://www.ifj.org/media-centre/news/detail/category/press-releases/article/cambodia-parliament-approves-state-emergency-law-amid-the-pandemic.html. Accessed 8 July 2020.

[CR18] Kagumire, Rosebell. 2020. Fighting a war or a virus? How blanket Covid-19 measures hit the poor. https://www.fes.de/en/africa-department/more-posts/fighting-a-war-or-a-virus-how-blanked-covid-19-measures-hit-the-poor. Accessed 14 July 2020.

[CR19] Maerz, Seraphine F., Anna Lührmann, Sebastian Hellmeier, Sandra Grahn, and I. Lindberg Staffan. 2020. State of the world 2019: autocratization surges—resistance grows. *Democratization*10.1080/13510347.2020.1758670.

[CR20] Metternich, Nils W. 2020. Drawback before the wave? Protest decline during the COVID-19 pandemic. https://osf.io/preprints/socarxiv/3ej72. Accessed 25 June 2020.

[CR21] Nasr, Joseph. 2020. Germany have right to protest during Coronavirus pandemic: court. *Reuters, *16 April 2020. https://www.reuters.com/article/us-health-coronavirus-germany-protests/germans-have-right-to-protest-during-coronavirus-pandemic-court-idUSKCN21Y220. Accessed 8 July 2020.

[CR22] OHCHR (Office of the UN High Commissioner for Human Rights). 2020. COVID-19: states should not abuse emergency measures to suppress human rights—UN experts. https://www.ohchr.org/EN/NewsEvents/Pages/DisplayNews.aspx?NewsID=25722. Accessed 6 July 2020.

[CR23] Poppe, Annika E., and Jonas Wolff. 2017. The contested spaces of civil society in a plural world: norm contestation in the debate about restrictions on international civil society support. *Contemporary Politics* 23(4):469–488.

[CR24] Rutzen, Doug, and Nikhil Dutta. 2020. Pandemics and human rights. *Just security*. https://www.justsecurity.org/69141/pandemics-and-human-rights. Accessed 6 July 2020.

[CR25] Safwan, Luna. 2020. Is the Lebanese government using COVID-19 to undermine protests? https://timep.org/commentary/analysis/is-the-lebanese-government-using-covid-19-to-undermine-protests. Accessed 8 July 2020.

[CR26] UN (United Nations). 1966. International covenant on economic, social and cultural rights. https://www.ohchr.org/en/professionalinterest/pages/cescr.aspx. Accessed 6 July 2020.

[CR27] Walker, Shaun. 2020. Hungary Passes Law that Will let Orbán Rule by Decree. *The Guardian, *30 March 2020. https://www.theguardian.com/world/2020/mar/30/hungary-jail-for-coronavirus-misinformation-viktor-orban. Accessed 8 July 2020.

